# Structural Similarities, in Relation with the Cross-Reactivity, of Hymenoptera Allergenic Dipeptidyl Peptidases IV—An Overall Comparison Including a New Dipeptidyl Peptidase IV Sequence from *Vespa velutina*

**DOI:** 10.3390/toxins15110656

**Published:** 2023-11-14

**Authors:** Rafael I. Monsalve, Manuel Lombardero, Lars H. Christensen, Beatriz Núñez-Acevedo, David González-de-Olano, Miriam Sobrino-García, Rosita M. Castillo-Loja, Susana B. Bravo, Manuela Alonso-Sampedro, Carmen Vidal

**Affiliations:** 1 Research and Development, Alk-Abelló A.S., 28037 Madrid, Spain; mlves@alk.net; 2 Global Research, ALK-Abelló A/S., 2970 Hørsholm, Denmark; larsharder.christensen@alk.net; 3 Allergology Service, Hospital Universitario Infanta Sofía, San Sebastián de los Reyes, 28702 Madrid, Spain; bnunezacevedo@yahoo.es; 4 Allergy Service, Hospital Ramón y Cajal, IRYCIS (Instituto Ramón y Cajal de Investigación Sanitaria), 28034 Madrid, Spain; dgolano@yahoo.es; 5 Allergology Service, Hospital Universitario de Salamanca, 37007 Salamanca, Spain; miriamsobrino@saludcastillayleon.es; 6 Biosanitary Institute, IBSAL (Instituto de Investigación Biomédica de Salamanca), 37007 Salamanca, Spain; rcastilloloja@usal.es; 7 Proteomic Unit, Health Research Institute of Santiago de Compostela (IDIS), University Hospital of Santiago de Compostela, 15706 Santiago de Compostela, Spain; sbbravo@gmail.com; 8 Research Methods Group (RESMET), Health Research Institute of Santiago de Compostela (IDIS), Network for Research on Chronicity, Primary Care, and Health Promotion (RICAPPS-ISCIII/RD21/0016/0022), University Hospital of Santiago de Compostela, 15706 Santiago de Compostela, Spain; manuela.alonso.sampedro@sergas.es; 9 Allergy Department, University Hospital of Santiago de Compostela, 15706 Santiago de Compostela, Spain; carmen.vidal.pan@sergas.es

**Keywords:** *Vespa velutina*, DPPIV, allergen, cross-reactivity, Hymenoptera, structure

## Abstract

(1) Background: Dipeptidyl Peptidases IV (DPPIVs), present in many organisms, are minor components in the venoms of Hymenoptera, where they have been identified as cross-reactive allergenic molecules. Considering that the structure of homologous DPPIVs is well characterized, we aimed to explain which regions have higher similarity among these proteins and present a comparison among them, including a new *Vespa velutina* DPPIV sequence. Moreover, two cases of sensitization to DPPIVs in wasp- and honeybee-sensitized patients are presented. (2) Methods: Proteomic analyses have been performed on the venom of the Asian hornet *Vespa velutina* to demonstrate the sequence of its DPPIV (allergen named Vesp v 3, with sequence accession number P0DRB8, and with the proteomic data available via ProteomeXchange with the identifier PXD046030). A comparison performed through their alignments and analysis of the three-dimensional structure showed a region with higher similarity among Hymenoptera DPPIVs. Additionally, ImmunoCAP™ determinations (including specific inhibition experiments), as well as IgE immunoblotting, are performed to demonstrate the allergenicity of Api m 5 and Ves v 3. (3) Results and Conclusions: The data presented demonstrate that the similarities among Hymenoptera DPPIVs are most likely localized at the C-terminal region of these enzymes. In addition, a higher similarity of the *Vespa*/*Vespula* DPPIVs is shown. The clinical cases analyzed demonstrated the allergenicity of Api m 5 and Ves v 3 in the sera of the allergic patients, as well as the presence of this minor component in the preparations used in venom immunotherapy.

## 1. Introduction

Dipeptidyl Peptidases IV (DPPIVs) are type II transmembrane serine proteases, also known as CD26 or adenosine deaminase binding protein [1]. As enzymes present in a vast number of organisms (from humans to invertebrates), DPPIVs are known to act as major regulators of various physiological processes in humans, including immune, inflammatory, nervous, and endocrine functions [2]. DPPIVs in Hymenoptera are also active enzymes, existing as minor components of their venom [3,4], but considered a major allergen due to the high number of patients sensitized to these proteins in wasps and honeybees [3,4,5], also recently described in the venom of the Asian hornet, *Vespa velutina* [6,7]. The physiological role of DPPIVs in the *Vespa* genera, and probably in other Hymenoptera, seems related to the maturation of the lytic toxin melittin or that of mastoparan B [7,8].

Human DPPIV has been structurally very well characterized [1,2], and it is known to be a glycoprotein with multiple N-glycosylation sites, even though it has been determined that glycosylation does not contribute significantly to its peptidase activity and that its carbohydrates do not affect dimerization (a key issue for its peptidase activity, as mentioned in [2]). Regarding Hymenoptera DPPIVs, their allergenicity has been shown to clearly depend on the protein backbone [3,5] in spite of the reactivity of CCDs, which always needs to be taken into account when performing “in vitro” determinations [4].

DPPIVs are considered responsible for cross-reactivity among Hymenoptera venoms [3,4,5,6,7], even if only one component present in the venom of honeybees, rApi m 5, is available as a reagent in ImmunoCAP™ for “in vitro” determinations [9,10,11]. Since rApi m 5 was initially tested as a honeybee venom component, it was demonstrated as a major allergen, with a prevalence of 58.3% (84 out of 144 patients). Its cross-reactivity with wasp allergic patients was shown in 3 out of 40 cases, being the only cross-reactive allergen responsible for double sensitization *Apis*/*Vespula*, apart from the hyaluronidases, due to the similarities between Api m 5 and its counterpart in *Vespula* spp., named Ves v 3 [10]. The component rApi m 5 has the advantage of not being affected by cross-reactivity attributable to CCDs (cross-reacting carbohydrates), but only depending on antigenic determinants of the protein backbone [10,11]. This cross-reactivity had already been indicated by Blank et al. [4], as well as being shown for *Polistes dominula*’s Pol d 3 [5] and for the DPPIV present in hornets [6,7,9] as is the case of *Vespa velutina*, the invasive Asian hornet that has spread over several European countries [6,12,13]. In the case of the Asian hornet, the complete sequence of the mature DPPIV, the allergenic Vesp v 3 [14,15], is presented in this manuscript.

## 2. Results

### 2.1. Structural Similarities in DPPIVS among Hymenoptera Species

The following sections describe a new DPPIV sequence, the similarities among Hymenoptera DPPIVs and several structural features of these proteins, in relation with their cross-reactivity.

#### 2.1.1. New Complete Vespa velutina Sequence and Comparison with Vespidae and Apidae DPPIVs

**Figure 1:**
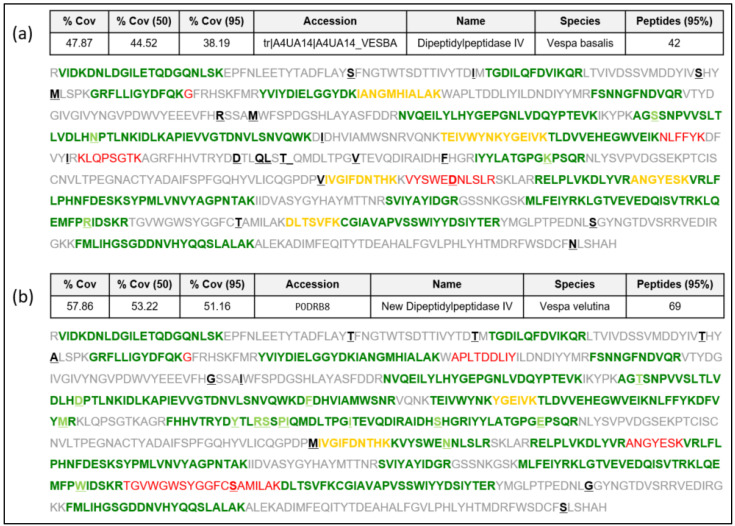
**New Vesp v 3 sequence, showing the peptides found using mass spectrometry (MS/MS).** Protein sequencing using ProteinPilot (sciex): (**a**) A search using UniProtKB database including *Vespa basalis* (accession number A4UA14), which was the only *Vespa* DPPIV protein sequence known to date. (**b**) Search using the new Vesp v 3 protein sequence, with the newly assigned accession number P0DRB8. The different amino acids between the sequences are enhanced in bold and underlined. Green peptides are identified with 95% confidence, yellow peptides are identified with a confidence between 50 and 95%, and red peptides have < 50% of confidence. Sequences shown in grey correspond to the part of the proteins not identified with MS. The data presented as “%Cov” refers to the “Coverage” the total regions identified with MS at the different levels of confidence; in each case, the number of peptides identified with 95% confidence is shown.

Sequence data extracted from the *Vespa velutina* transcriptome [16] as indicated in [17] are presented in this manuscript as a new DPPIV sequence in the Hymenoptera species (named “Vesp v 3” as it corresponds to this allergenic molecule [4,7,14,15]. We present the complete confirmatory proteomic data of the DPPIV protein identified in *Vespa velutina* venom preparations from insects in the north-west regions (Galicia, Spain) of the prepared Spanish peninsula, as described in [18] and detailed in the Materials and Methods. The data presented in Figure 1b show strikingly high coverage of the sequence corresponding to this sequence (57.9% overall coverage), as determined by LC MS/MS on different preparations of *Vespa velutina* venom, with 69 peptides identified in the analyses. The newly confirmed Vesp v 3 sequence will be available in the UniProtKB database [19] with the accession number “P0DRB8”. The proteomic data in Figure 1a also show the peptides that would match with the only *Vespa* DPPIV sequence described to date (the sequence from *Vespa basalis*, with accession number A4UA14; [8]), for which only 42 peptides are identified, with a lower coverage (47.9% overall coverage) than for the *Vespa velutina* DPPIV. Data in Figure 1 show that the *Vespa basalis* DPPIV is similar to the Vesp v 3 sequence, but it is not the specific sequence determined in this manuscript. The alignment shown in Figure S1 (Supplementary Materials) also shows their similarity (97% sequence identity). A detailed analysis of the proteomic data is shown in Figures S2 and S3 of the Supplementary Materials, presented as an appendix to this manuscript. Moreover, the complete proteomic data are deposited in the PRIDE partner repository with the dataset identifier PXD046030 [20].

**Figure 2:**
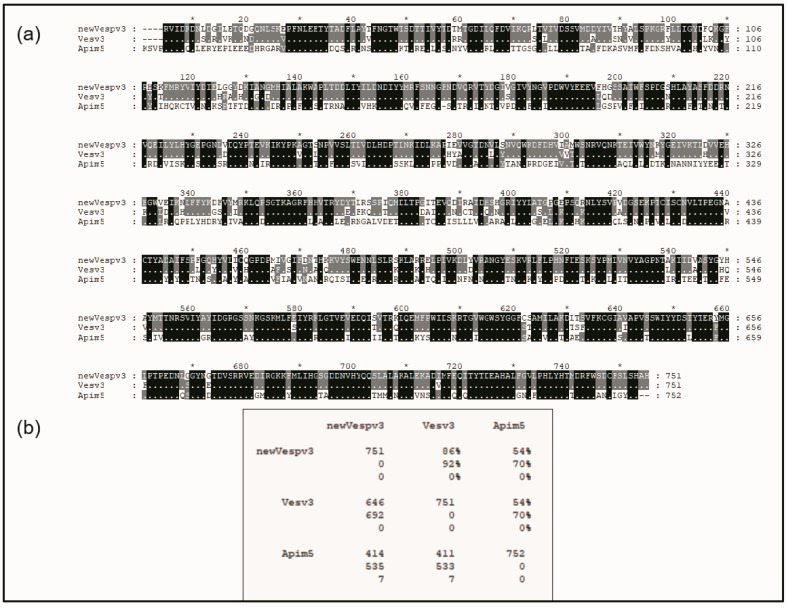
**Sequence alignment of the mature DPPIV sequences from Hymenoptera.** The sequences compared correspond to the mature proteins from *Vespa velutina* (accession number P0DRB8; shown as “new Vespv3”), *Vespula vulgaris* (accession number B1A4F7, shown as Vesv3), and from *Apis mellifera* (accession number B2D0J4, shown as Apim5). Panel (**a**) shows the multiple alignment editing prepared with GeneDoc software [21] that shows the numbering for the Vesp v 3 sequence on the top of the aligned sequences (with an “*” that facilitates the reading of every tenth number), as well as the numbering order of each sequence at the right-hand side of each line. The top sequence (Vesp v 3′s) is completely visible, and when the amino acids for Ves v 3 and Api m 5 are shown, this is because they differ from the top sequence. The black dotted shading in the bottom sequences indicates complete identity among the three proteins compared, and grey shading indicates that there is identity between two of the sequences. Panel (**b**) shows the Comparison Matrix from the alignments shown, where the numbers exhibiting “%” values correspond to “total identity” (value on top) or “similarity” between the proteins (value immediately below), considering the conservative amino acids [22]. The numbering at the left part of the matrix refers to the total number of amino acids per protein (in the diagonal), or, when the numbers show the pair-to-pair comparison of the sequences, the top number corresponds to the “total identical residues”, the middle number to the “total similar residues”, and the bottom number to the “gaps” that had to be added to the alignment to display the best evolutionary alignment possible.

The mature sequence of Vesp v 3 and its alignment with the counterparts from *Vespula vulgaris* and *Apis mellifera* are shown in Figure 2. The sequence identities of Vesp v 3 and Ves v 3 are very high (86% identity and 92% similarity), indicating the high degree of similarities among the sequences of the *Vespa* and *Vespula* genera. Very high similarity values (93% sequence identity) were also found among the hyaluronidases of these two genera, as shown by Monsalve et al. [17]. The DPPIV similarity between the Vespinae genera (*Vespula* and *Vespa*) and *Polistes* is much lower, with 76.6% sequence identity [5].

Sequence similarities between wasps and hornet DPPIVs with Api m 5 have much lower values, with 54% sequence identity, as can be observed in the overall alignment shown in Figure 2. This very low sequence similarity very weakly supports the reported cross-reactivity existing among vespids and honeybees. Nevertheless, a closer look into the protein sequence alignments in Figure 2 also clearly shows a region of higher sequence similarity at the C-terminal part of the sequences: there are clearly many more “black rectangles” after position #555 of the aligned sequences in Figure 2, indicating many regions of complete identity among the three proteins aligned, as will also be described in the next section.

#### 2.1.2. Similarities with Other DPPIV Enzymes: A Conserved Domain of the DPPIVs

**Figure 3:**
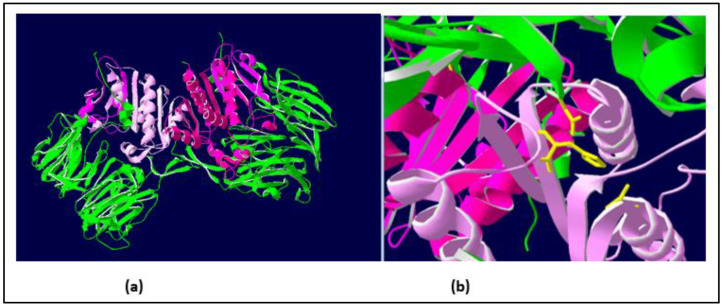
**Three-dimensional (3D) structure of human DPPIV showing the two structural domains of these enzymes**. Coloring of the molecules has been prepared by R.I.Monsalve, using the Swiss-PdbViewer program [24]. Panel (**a**) (**left**): The 3D structure of human DPPIV heterodimer [1], in which the two domains of the molecules are shown in green (N-terminal domain) and purple (C-terminal domain, beginning at position #555 in the sequence shown in Figure 2). Panel (**b**) (**right**): detail of the “catalytic triad” responsible for the enzymatic activity of DPPIV (showing the residues in yellow).

**Figure 4:**
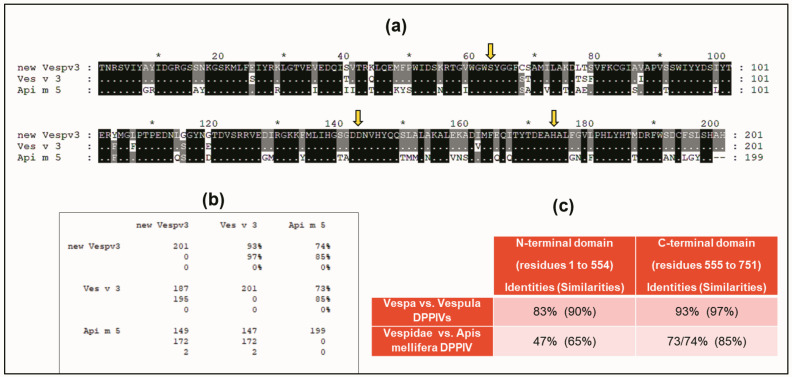
**Sequence alignment of sequences at the C-terminal domain of Hymenoptera DPPIVs.** Alignment of C-termini of Vesp v 3/Ves v 3/Api m 5, and identity/similarity matrix (using the same alignment scheme and symbols as explained in Figure 2). Yellow arrows indicate the amino acids that constitute the “catalytic triad” of all DPPIVs (serine, aspartic acid, and histidine, which are also shown in yellow in panel b of Figure 3). Panel (**b**) shows the identity/similarity matrix of the three sequences compared. Panel (**c**) shows a comparison of the similarities found in the separate alignments of the two domains: the C-terminal shown in panel (**a**) and the alignment of the N-terminal domain (amino acids 1 to 554 according to the numbering of the whole mature sequence of Vesp v 3 as shown in Figure 1). The data corresponding to the new sequence for Vesp v 3 are shown as “new Vespv3” in panels (**a**,**b**).

A BLAST sequence alignment [23] of the Vesp v 3 sequence, apart from the homologous Hymenoptera counterparts, also found sequence similarities with other DPPIV (e.g., 32% sequence identity with the Human-DPPIV, UniProtKB/Swiss-Prot, accession number P27487 [4]). They are indeed homologous proteins, and it has already been described that all DPPIV enzymes are constituted by two domains, an N-terminal dipeptidyl peptidase domain and a C-terminal prolyl peptidase domain, as also indicated when Ves v 3 and Api m 5 were identified as relevant allergens [4]. Moreover, the three-dimensional (3D) structure of human DPPIV is also available (PDB reference: Apo dipeptidyl peptidase IV/CD26, 1pfq), and it has therefore been extensively characterized [1,2]. The specific coloring shown in Figure 3 (prepared by R.I. Monsalve using the Swiss-PdbViewer program for [24]) clearly differentiates the two structurally independent domains of these DPPIVs. The sequences corresponding to the purple-colored C-terminal domains, aligned for the Hymenoptera DPPIVs, are shown in Figure 4. As can be observed, the similarity among these proteins increases significantly in comparison with the overall alignment shown in Figure 2. The identity/similarity matrix and the data summarized in Figure 4b,c show a much higher similarity among the proteins compared at the C-terminal domain, and the most significant result indicates that *Vespa*/*Vespula* similarity to Api m 5 increases from 65% to 85% (from 47% to 73–74% if we consider sequence identity). This much higher similarity among these proteins at the C-terminal domain of the DPPIV provides a significantly increased chance of antigenic cross-reactivity among Hymenoptera DPPIVs, and this area of the DPPIV is more likely to contain common antigenic determinants. Panel c in Figure 4 shows that the similarity at the N-terminal domain of Hymenoptera DPPIVs is much lower than what occurs at the C-terminal end.

One additional confirmation of the structural similarity among DPPIVs in this particular domain is the fact that the amino acids responsible for the enzymatic activity of this domain (the “catalytic triad”, as indicated in [1]), are conserved in all of the Hymenoptera sequences studied, in addition to being present in the human DPPIV. These amino acids are shown in yellow in panel b of Figure 3, and they are also indicated with yellow arrows in the alignment of the C-termini of the Hymenoptera DPPIVs (Figure 4a). This high degree of conservation, in addition to confirming the 3D similarities of the Hymenoptera DPPIVs, also indicates an evolutionary restriction for the functionality of these enzymes, which has led to a strict conservation of this domain of the DPPIV from such diverse sources.

## 3. Clinical Cases of Specific Allergenicity of Hymenoptera DPPIV Components

Two clinical cases of Hymenoptera patients with peculiar allergenicity to DPPIV, detected by its reactivity to the ImmunoCAP™ component Api m 5, are presented in this section of the manuscript.

### 3.1. Ves v 3 Allergenicity Detected by Its Cross-Reactivity with Api m 5 in ImmunoCAP

**Figure 5:**
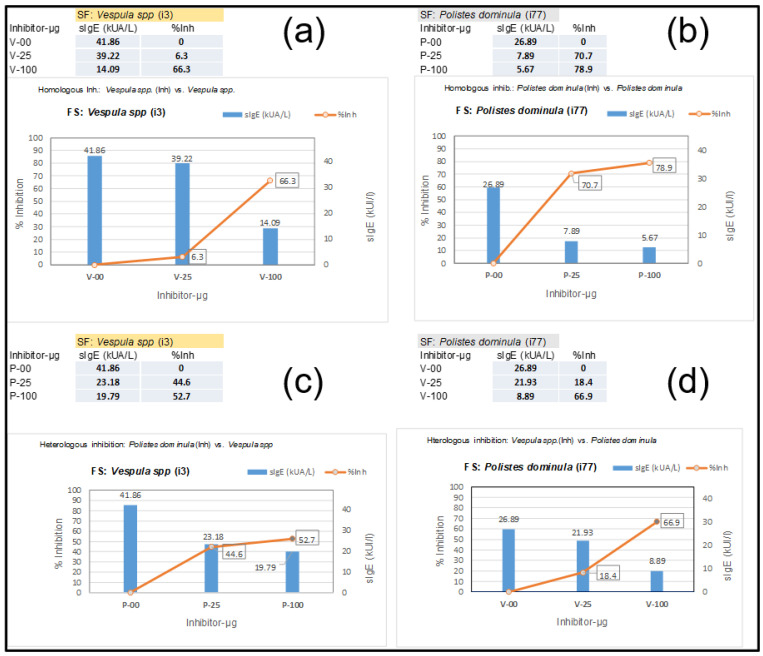
**ImmunoCAP inhibition studies with serum presented in Section 3.1.** Results of inhibition studies correspond to “Homologous inhibitions” (**a**,**b**) and reciprocal or heterologous inhibitions. (**c**) shows the inhibition by *Polistes dominula*s extract (0/25/100 µg) on *Vespula* spp. (i3) in the solid phase, and (**d**) shows the opposite (*Vespula* spp. extract inhibiting the sIgE in the i77 solid phase). The blue vertical bars represent the kU/L value for different dilutions, with the numerical value also shown. The numbers enclosed in black rectangles indicate the % of inhibition in each determination. These sIgE values and percentages (%inh) for each inhibition are also shown in the tables at the left side of the (**a**–**d**) labels.

A 44-year-old woman with previously reported extensive local reactions after Hymenoptera stings was urgently referred for an immediate response after an additional Hymenoptera sting by an unidentified insect with the following symptoms: palmoplantar pruritus, generalized urticaria, eyelid and labial angioedema, sensation of chest tightness, and dyspnea. She was immediately treated with adrenaline, methylprednisolone, and dexchlorpheniramine, and her symptoms disappeared within 12 h. After treatment, a complete allergologic study was performed, including the “in vitro” testing presented in this manuscript, including Component Resolved Diagnosis (CRD) and CAP-inhibition studies. Table 1 shows the specific IgE determinations of the extracts and diverse components that are relevant in this case, with an initial “basal serum” from the patient and additional measurements after the patient began VIT. Basal tryptase, before the patient started any venom immunotherapy (VIT) treatment, presented values of 4.83 µg/L. The sIgE values of the patient, before VIT, led us to perform CAP-inhibition studies [25,26] in order to decide on further treatment. These inhibitions are presented in Figure 5, showing the unusual results of the *Vespula*/*Polistes* inhibition studies. The inhibitions required a higher concentration than usual of the *Vespula* and *Polistes* extracts (up to 100 µg of the extracts were necessary) to achieve significant inhibition, even in the case of “homologous inhibitions” (Figure 5a,b), for which the inhibition with *Vespula* spp. required 100 µg of the extract to achieve a 66.3% inhibition (Figure 5a), after which the *Vespula* spp. values decreased from 41.86 to 14.09 KUA/L. In the case of the homologous inhibition of *Polistes dominula* (Figure 5b), with starting values of 26.9 KUA/L, the inhibition with 25 µg of extract was already above 70% (reducing the sIgE values versus the *Polistes dominula* solid phase to 7.89 KUA/L), and it increased further using 100 µg of the *Polistes dominula* extract (i.e., 79% inhibition).

The “reciprocal inhibitions” (Figure 5c,d) showed that the *Polistes dominula* extract only inhibited the *Vespula*-specific IgE in the solid phase up to 53%, even with the highest quantity of the *Polistes dominula* extract (Figure 5c). On the contrary, the *Vespula* spp. extract inhibited up to 66% of the sIgE toward *Polistes dominula*. The higher inhibitory strength of the *Vespula* spp. extract (aside from the highest sIgE values toward each of the extracts) indicated that the patient was primarily sensitized by *Vespula* species, and a VIT treatment with ALUTARD SQ^®^-*Vespula* spp. was started in July 2022. The evolution of the patient’s sIgE, total IgE, and IgG4 values is shown in Table 1. The data show that the sIgE values for the different components are clearly decreasing, indicating that the VIT seems to be progressing successfully.

Nevertheless, the unusual data shown in the CAP-inhibition studies (especially the high concentration of the *Vespula* spp. extract needed to obtain a clear inhibition of the sIgE) revealed the significant relevance of minor components of the venom extract. As a matter of fact, this patient showed a very unusual sIgE value toward the Api m 5 component in ImmunoCAP™ (54.9 KAU/L as shown in Table 1 in the initial analyses of the patient’s serum before starting VIT). The apparent irrelevance of sensitization to *Apis mellifera*, considering the complete clinical history of the patient, in conjunction with the “peculiar” CAP-inhibition results, led us to think about this relevant influence on the homologous DPPIV component in *Vespula* spp. venom. Indeed, the possible cross-reactivity to Ves v 3 would explain the peculiar results mentioned previously. This idea can be further corroborated by the sIgE-immunoblots presented in Section 3.3.

In any case, the data shown in Table 1, with regard to the sIgE reaction to the rApi m 5 component, also indicate that the ongoing VIT was proceeding successfully (after 6 months and 12 months of treatment, the sIgE values to Api m 5 have decreased to 27.4 and 14.5 KAU/L, respectively). On the other hand, the measurements of IgG4 against the *Vespula* spp. extract and the Api m 5 component (Table 1) still did not show any increase, probably due to the fact that it is still too early to observe such immunological changes, as indicated in [27,28], but the patient will be followed up in this regard.

### 3.2. Patient Predominantly Sensitized to Api m 5

Serum from a patient who suffered three syncopal episodes and sphincter relaxation after suffering bee stings was analyzed using ImmunoCAP-250, and the initial “in vitro” determinations showed the following sIgE values (KAU/L): 2.75 for *Apis mellifera* extract (i1), 0.19 for rApi m 1 (i208), 0.0 for rApi m 2 (i214), 0.01 for rApi m 3 (i215), 0.0 for Api m 4 (h3x as ALK reference), and 0.14 for rApi m 10 (i217). Considering these generally very low or negative sIgE values, we also tested additional components with ImmunoCAP with the following results: 2.54 for rApi m 5 (i216), 0.28 for *Vespula* spp. Ext. (i3), 0.05 for rVes v 1 (i211), and 0.28 rVes v 5 (i209); the total IgE was 15 kU/L. These results provide insights into a predominant sensitization to Api m 5 in this *Apis mellifera*-sensitized patient since this was the only component showing clear sIgE positivity, with a similar value to that of the whole *Apis mellifera* extract. Moreover, after a complete hematological study including a bone marrow biopsy, the patient was diagnosed with bone marrow mastocytosis (the basal serum tryptase had values of 9.16 ng/mL).

**Figure 6:**
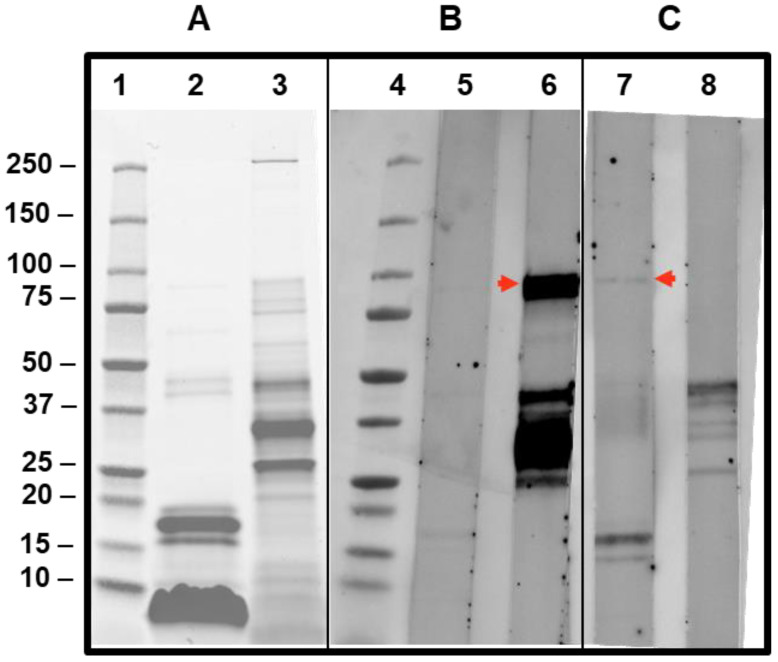
SDS-PAGE and IgE immunoblotting of patients’ sera against *Apis mellifera* and *Vespula* spp. extracts. SDS-PAGE and IgE immunoblotting show the results of testing for 15 µg of *Apis mellifera* extract (lanes 2, 5, and 7) and *Vespula* spp. extract (lanes 3, 6, and 9) in SDS-PAGE-Oriole staining (panel (**A**) after transferring to nitrocellulose membranes and showing IgE-immunoblotting on the sera from the patient described in Section 3.1 (panel (**B**), lanes 5 and 6) and the patient described in Section 3.2 (panel (**C**), lanes 7 and 8). Lanes 1 and 4 show the molecular weight ladder (BIO-RAD Precision Plus standards) of the sizes in kDa indicated at the left of panel A. The red arrows intend to show the IgE-recognized Ves v 3 (lane 6) and Api m 5 (lane 7), as discussed in the text.

Despite the very low sIgE values, CAP-inhibition studies were also performed in this case, and inhibitions of 38.3 and 46% were achieved on the sIgE for Api m 5 when 25 and 100 µg, respectively, of AquagenSQ^®^-*Apis mellifera* extracts were used. These data confirm the relevance in this patient of this minor component of the *Apis mellifera* venom. The low abundance of Api m 5 was clearly shown in the SDS-PAGE and IgE-immunoblot (Figure 6), being barely visible in the *Apis mellifera* extract in the SDS-PAGE (lane 2), and the sIgE reactivity toward Api m 5 is weak (Figure 6, lane 7) in comparison with the Api m 1 response, in spite of the higher values on ImmunoCAP™ for Api m 5 (2.54) in comparison with the very low value measured for Api m 1 (0.19). Honeybee A2-phospholipase (Api m 1) is a clear major component of this venom, as also shown in the SDS-PAGE result shown in Figure 6 (lane 2, Api m 1 being the strongly stained bands around 17 kDa).

This patient began VIT with ALUTARD SQ^®^-*Apis mellifera* since December 2022, and after five months, the IgG4 values for *Apis* increased from 0.35 to 1.54 mgA/L, while no increase had yet been detected for the Api m 5 component. Nevertheless, these measurements were still premature [27,28], and the patient’s evolution will be followed up regarding sIgG4 values and other general immunological parameters.

Moreover, as is shown in the next section, IgE-immunoblotting has also demonstrated a specific Api m 5 sensitization in this *Apis*-sensitized patient (Figure 6), in addition to a clear reactivity toward the major venom component Api m 1.

### 3.3. Immunoblot of Patient’s Sera Showing Positivity to DPPIV Components in Vespula spp. and Apis Mellifera Extract

Sera from the patient cases presented in this manuscript have been tested in IgE-immunoblotting experiments to observe the specificity in each case (Figure 6). The case of the patient described in Section 3.1 exhibits a very strong reactivity to a band that corresponds to Ves v 3 (lane 6, Figure 6), in addition to other allergenic components of the *Vespula* spp. extract tested, as expected from the sIgE values detected with this serum (Table 1). The strong reactivity of an 88–90kDa protein is compatible with this Ves v 3 reactivity (for the *Apis* extract, the Api m 5 cross-reactive band is also visible, albeit with a much weaker intensity, confirming the wasp sensitization of the patient).

The results of the patient’s case described in Section 3.2 (lane 7 in Figure 6) also show, in addition to the recognition of a 17kDa band corresponding to the major component Api m 1 in the *Apis mellifera* extract, a positive response around the 90kDa band (red arrow shown in lane 7 of Figure 6), confirming the sensitization of the patient studied to Api m 5, in spite of the low value for sIgE measured using ImmunoCAP. This confirms IgE reactivity to the polypeptide antigenic determinants of this *Apis mellifera*-DPPIV.

## 4. Discussion

In this manuscript, we presented the complete sequence data of the mature DPPIV component present in *Vespa velutina*, with the assigned UniProtKB accession number P0DRB8, also recently described as a relevant allergenic component of the venom of this species [6,7] and therefore named Vesp v 3 [14,15]. We compared this new sequence with that of other Hymenoptera DPPIVs (Figure 2). *Vespa* and *Vespula* genera exhibit high similarity values (reaching values of 92%), with *Apis* being more dissimilar (70% similarity). The similarity within the *Vespa* genera is also very high: 97% sequence identity was found between the DPPIVs of *Vespa basalis* [8] and the new sequence from *Vespa velutina*.

On the other side, a detailed consideration of the 3D organization of DPPIVs has allowed us to show that in one of the two domains that constitute these enzymes, a significantly higher similarity between *Vespula*/*Vespa* and *Apis* is observed (increasing to 85% in the similarity values shown in Figure 4c, while their similarity at the N-terminal domain only reaches 65%). The highest similarity in these conserved domains of DPPIVs makes it much more probable to explain the cross-reactivity among Hymenoptera DPPIVs. The exact localization of the common antigenic determinants requires additional studies to elucidate which area is responsible for the cross-reactivity existing between these proteins. These studies will be feasible, considering the availability of recombinant DPPIVs and the detailed knowledge on the three-dimensional structure of DPPIVs [1,2], as well as the availability of recombinant monoclonal antibodies that recognize specific epitopes [29].

In this paper, we also provided examples of the already-mentioned cross-reactivity [3,4,5,6,7,9,30] between the DPPIVs of honeybees and wasps, with one demonstrated case of cross-reactivity of the Ves v 3 component (Section 3.1), revealed in ImmunoCAP-inhibitions studies, and also shown in the specific detection of Ves v 3 in immunoblot experiments (Figure 6, lane 6), with Ves v 3 being the homologous counterpart of Api m 5 [4]. These CAP-inhibition studies were, again, extremely useful for determining the specific sensitizations of patients, as previously shown in other studies in which *Vespula*/*Polistes* double positivity was resolved [25,31,32], and in this manuscript, they were also shown to be useful in particular cases in which minor components of the venoms have a relevant influence on the sIgE values. As a matter of fact, we have dealt with a very special case (presented in Section 3.1 and Section 3.3) in which a *Vespula/Polistes* CAP-inhibition case did not meet the usual behavior for these experiments [26,31]. As shown in Figure 5a,b, the homologous inhibitions required unusually high concentrations of the inhibiting *Vespula* spp. extract (100 µg) to achieve an inhibition of 66.3% (Figure 5a), while the *Polistes dominula* extract behaved as expected for the majority of cases [25,26,31], with an inhibition above 70% with only 25 µg of the *Polistes dominula* inhibiting extract (Figure 5b) and reaching 79% when 100 µg was used. This peculiar case, which was finally considered a *Vespula* spp.-sensitized patient, considering the poor inhibition of the *Polistes dominula* extract (only 52.7% with 100 µg of inhibiting extract, Figure 5c), compared with the 67% inhibition of *Vespula* spp. on the sIgE to *Polistes dominula* in the solid phase (Figure 5d), demonstrates a specific case in which minor components, as in the case of Ves v 3, have a relevant influence. The immunoblotting shown in Figure 6 (lane 6) also confirms the specific reactivity of the Ves v 3 component, and the results presented in Table 1 also suggest that the patient is receiving the correct VIT, according to the evolution of the overall immunological parameters.

We also presented a case (Section 3.2) of a patient predominantly sensitized to Api m 5. In this particular case, there was a clear ImmunoCAP inhibition with Aquagen SQ^®^-*Apis mellifera* on the sIgEs toward Api m 5, with this fact being especially interesting since it demonstrated the presence of this minor component of the venom of *Apis mellifera* in this preparation, contradicting what had been mentioned with regard to its absence in the immunotherapeutic preparation [33]. The patient is being treated with ALUTARD SQ^®^-*Apis mellifera*, a pharmaceutical product available through the Spanish National Health System, which must also contain Api m 5. As a matter of fact, these two immunotherapeutic products, both in their aqueous and depot preparations, contain the same components of the venom because they are prepared following the same purification scheme, as also indicated by Bilo et al. [34].

Moreover, the IgE immunoblots shown in Section 3.3 demonstrate the presence of allergenic proteins with an apparent molecular weight of around 90kDa, which must correspond to the protein backbones of the homologs Ves v 3 and Api m 5 (lanes 6 and 7, respectively, in Figure 6). The precise regions of antigenic cross-reactivity remain to be elucidated, as previously mentioned. The results of the two clinical cases described in this manuscript demonstrate the importance of testing for the Api m 5 component in ImmunoCAP, both in cases of patients sensitized exclusively to honeybees and in cases of suspected *Apis*/*Vespula* double sensitization.

Additional evidence with patients sensitized to other Hymenoptera DPPIV components is needed to better understand the demonstrated cross-reactivity observed among Hymenoptera, but the very high similarities existing at the C-terminal domain of these proteins may allow for the elucidation of the specific regions responsible for the cross-reactivity among different DPPIVs.

## 5. Material and Methods

### 5.1. Proteomic Studies

#### 5.1.1. Venom Collection

The *Vespa velutina* nest was collected in Galicia (NW Spain) during the winter of 2022 and was stored at −20 °C until it was used in the study. After sex and female caste differentiation, based on external morphological characteristics, the venom sacs were extracted from frozen insects by pulling the stinging apparatus from the wasp abdomen with forceps. The venom sacs of 32 workers were dissected from their posterior apparatus. Eight venom sacs were then pooled, resulting in a total of four pooled samples, representing four biological replicates (named VV 1 to VV 4). The pooled samples were then eluted through a Spin-X 0.45 µm cellulose acetate centrifuge filter (Corning Inc., Salt Lake City, UT, USA), and the residual tissue from each venom sac was removed via centrifugation at 14,000 rpm for 10 min. The Spin-X was then washed with 250 µL PBS (phosphate-buffered saline) and centrifuged at 3000 rpm for 3 min. The eluted total venom protein was transferred to a new tube and stored at −20 °C. Total venom proteins were quantified using the Bradford method (Bio-Rad, Hercules, CA, USA).

#### 5.1.2. Mass Spectrometric Analyses

The tryptic digestion for mass spectrometry (MS) was performed with 24 µg protein concentrated in an SDS-PAGE single band [35,36], followed by manual digestion as described in [37]; finally, the peptides extracted were dissolved in 0.1% formic acid for further analysis.

Mass spectrometry analysis was performed using a hybrid quadrupole-TOF mass spectrometer 6600+ (Sciex, Framingham, MA, USA) coupled to an Ekspert nLC425 micro-liquid chromatography (LC) system (Eksigen, Dublin, CA, USA). ProteinPilot software v.5.0.1. (Sciex, Framingham, MA, USA) was used for protein and peptide identification. A customized database containing *Vespa* + *Vespa velutina* + *Apis mellifera* + poison + toxins UniProtKB databases available online (https://www.uniprot.org/ (accessed on 2 February 2023)) was used, specifying iodoacetamide as alkylation of the cystines and digestion by trypsin. A pool was created using 3 µL of the peptides extracted from each individual sample (VV 1–4). The pool was chromatographed for a total time of 40 min and analyzed using a data-dependent acquisition (DDA) method in a positive ion mode to build the MS/MS spectral libraries, as previously described [38,39,40]. The false discovery rate (FDR) was set to 1% for both peptides and proteins, with a confidence level above 99% [41]. The MS/MS spectra, ion data, and retention time of the identified peptides and proteins were used to generate the spectral library that was used to create the spectral window acquisition used in the SWATH-MS method. Subsequently, 4 µL from each sample was analyzed individually. The SWATH-MS method (DIA: data-independent acquisition) is based on the repetition of a cycle consisting of the acquisition of 100 TOF MS/MS scans, or time-of-flight mass spectrometry (TOF MS/MS) windows. The width of the variable windows was optimized for each set of samples according to the ion density found in the previous DDA via the SWATH variable window calculator (Sciex, Framingham, MA, USA) spreadsheet.

Skyline v.3.7 was used to process the DDA and DIA data files. The new DPPIV sequence was used to generate the best monoisotopic peak of each precursor ion. This peak was extracted using the software’s default settings for MS1 quantification. In addition, the best transitions for MS2 of each precursor were also selected among the DIA files. After importing the raw data into the Skyline software, the data files were queried against the protein sequence; peptide and fragment signals were manually checked to ensure correct peak assignment and peak boundaries. Those peptides with an amino acid different from the previously known *Vespa basalis* sequence were selected to better define and confirm the new peptide sequence.

The mass spectrometry proteomics data have been deposited to the ProteomeXchange Consortium via the PRIDE [20] partner repository with the dataset identifier PXD046030.

The sequence for the *Vespa velutina* DPPIV was obtained, as indicated in [17], from the publicly available RNA-seq data derived from *Vespa velutina* venom glands [16]. The ClustalW program [42] was used for performing multiple sequence alignments, and Swiss-PdbViewer was used to represent the three-dimensional (3D) structures [24]. Homologous proteins were found by BLAST searches in the public sequence databases [23].

### 5.2. SDS-PAGE and Immunoblotting

SDS-PAGE was performed on 4–20% acrylamide BIO-RAD’s Mini-Protean TGX Stain-Free gels. Immunoblots with patient sera were performed after transferring to nitrocellulose membranes using Trans-Turbo Blot (BIO-RAD) equipment. Serum was diluted 1/5, and specific IgE was detected using a mouse monoclonal anti-human IgE [43]; finally, the fluorescent StarBrightBlue700 GAM IgG (BIO-RAD) was used. Images from immunoblot or SDS-PAGE (after staining with Oriole™Fluorescent Reagent, BIO-RAD) were obtained using ChemiDoc MP (BIO-RAD) equipment.

### 5.3. Measurements on ImmunoCAP and CAP-FEIA Inhibition Assays

sIgE reactivity levels to the different components, as well as total IgE and IgG4-specific determinations, were obtained using ImmunoCAP-250 equipment (Thermofischer Sci., Waltham, MA, USA). Apart from several commercial components from Thermofisher, sIgE to Pol d 1 (purified as described in [44]) was also determined. Inhibitions on ImmunoCAP™ reactivity (named CAP-inhibition) were performed as indicated in [25,26] using quantities of 0, 25, or 100 micrograms of crude *Vespula* spp. venom, crude *Polistes dominula* venom (both from ALK-Source Materials, Post Falls, ID, USA), or Aquagen SQ^®^-*Apis mellifera* (from ALK-Abello A/S). The Aquagen preparation was specifically used for inhibition of the Api m 5 component with the indicated concentrations (0 µg for no inhibition, and 25 and 100 µg for progressive inhibition). In the case presented in Section 3.1, the same scheme is followed both for the “homologous” inhibition (*Vespula* spp. versus *Vespula* spp. in the solid phase and *Polistes dominula* versus *Polistes dominula* in the solid phase) and for the “reciprocal” inhibitions (*Polistes dominula* extract inhibiting *Vespula* spp. in the solid phase and *Vespula* spp. extract inhibiting *Polites dominula* in the solid phase). Patient serum and inhibitor extracts were preincubated at room temperature for 2 h prior to the assay in the ImmunoCAP-250. The extent of homologous and heterologous venom inhibition was calculated considering the decrease in sIgE value for the corresponding component in the solid phase (*Vespula* spp. extract, *Polistes dominula* extract, or the *Apis mellifera* component). Homologous inhibition was usually close to 100% using 25 µg of the pure extract, while inhibition values above 70% were considered an indication of extensive cross-reactivity [9,26,45].

## Figures and Tables

**Table 1:** **ImmunoCAP values for the poly-sensitized patient described in Section 3.1**. Specific IgE values against different components, as well as *Vespula* spp.-IgG4 and total-IgE, have been determined from the case described, with samples extracted before initiating immunotherapy (basal serum) or after six (6 M) or twelve (12 M) months of having started VIT.

	sIgE (kUA/L)											IgG4 (mgA/L)	IgE-Total (kU/L)
	*Ves v 1* *(i211)*	*Ves v 5* *(i209)*	*Pol d 1* (h6x*)	*Pol d 5* *(i210)*	*Vespula spp.* *(i3)*	*Polistes spp.* *(i4)*	*Apis mellifera* *(i1)*	*rApi m 1* *(i208)*	*rApi m 3* *(i215)*	*rApi m 10* *(i217)*	*rApi m 5* *(i216)*	*Vespula spp.* *(i3)*	*t-IgE*
***Basal serum***	**42.7**	**2.14**	**8.49**	**10.5**	**44.8**	**30.0**	**3.58**	**0.0**	**0.0**	**0.01**	**54.9**	*Ndet.*	**322**
***Serum (6M)***	**5.83**	**0.79**	**1.22**	**4.3**	**13.0**	*Ndet.*	4.18	*Ndet.*	*Ndet.*	*Ndet.*	**27.4**	**11.1**	**281**
***Serum (12M)***	**3.18**	**0.39**	**0.89**	**2.81**	**7.89**	*Ndet.*	*Ndet.*	*Ndet.*	*Ndet.*	*Ndet.*	**14.5**	**11.6**	**178**

* Component specifically purified and prepared by ALK-Abelló for ImmunoCAP determinations.

## Data Availability

The data presented in this work were made public by the authors by explicitly making public the new *Vespa velutina* sequence (UniProtKB database accession number P0DRB8), publishing the raw data in the Supplementary Material files and depositing the proteomic data generated into the PRIDE partner repository with the dataset identifier PXD046030.

## Supplementary Materials

The following supporting information can be downloaded at https://www.mdpi.com/article/10.3390/toxins15110656/s1. Figure S1 shows the alignment of the *Vespa basalis* DPPIV sequence, and on the newly presented *Vespa velutina* sequence, some specific differences important to the proteomic study are shown. Figures S2 and S3 show the detailed proteomic studies demonstrating the specific sequences of the new DPPIV *Vespa velutina* sequence. Three additional Figures (with no numbering) show the raw data used for the SDS-PAGE and immunoblot analyses presented in Figure 6.
