# Supplementary material for: Structural Similarities, in Relation with the Cross-Reactivity, of Hymenoptera Allergenic Dipeptidyl Peptidases IV—An Overall Comparison Including a New Dipeptidyl Peptidase IV Sequence from Vespa velutina
Source: Toxins (Basel). 2023 Nov 14;15(11):656. doi: 10.3390/toxins15110656 (PMC10675595; doi:10.3390/toxins15110656)

SUPPLEMENTARY MATERIAL to the manuscript

Structural Similarities, in Relation with the Cross-Reactivity, of Hymenoptera Allergenic Dipeptidyl Peptidases IV— An Overall Comparison Including a New Dipeptidyl Peptidase IV Sequence from *Vespa velutina*

Figure S1 -- Comparison of the DPPIV sequences. A red rectangle marks the different amino acids. Green peptides are identified with a confidence of 95%, yellow peptides are identified with a confidence between 50 and 95%, and red peptides have <50% confidence.

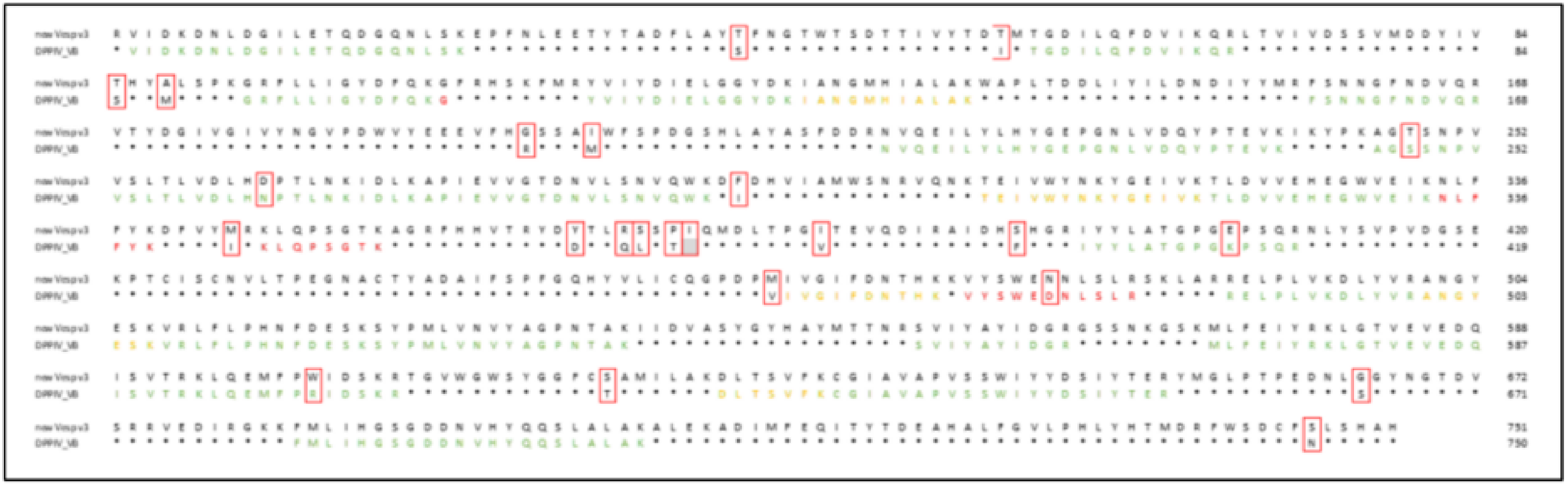

Figure S2 -- **Skyline DDA data.** A representative extracted precursor signal for the 4 peptides precursors K.AGTSNPVVSLTLVDLHDPTLNK.I , R.IYYLATGPGEPSQR.N, K.LQEMFPWIDSK.R, K.VYSWENNLSLR.S from DDA data acquired on VV samples digest using trypsin. The vertical lines on either side of the peak indicate the integration boundaries for the peak. The vertical blue line shows the retention time of the peptide identification contained in the protein sequence. The mass measurement error and retention time of the isotopic peak (precursor data) for the peptide precursor are annotated above the chromatographic peak. Amino acids shown in green correspond to residues that differ in the newVespv3 sequence and the sequence from *V.basalis*. DDA, data-dependent acquisiton; VV, *Vespa velutina.*

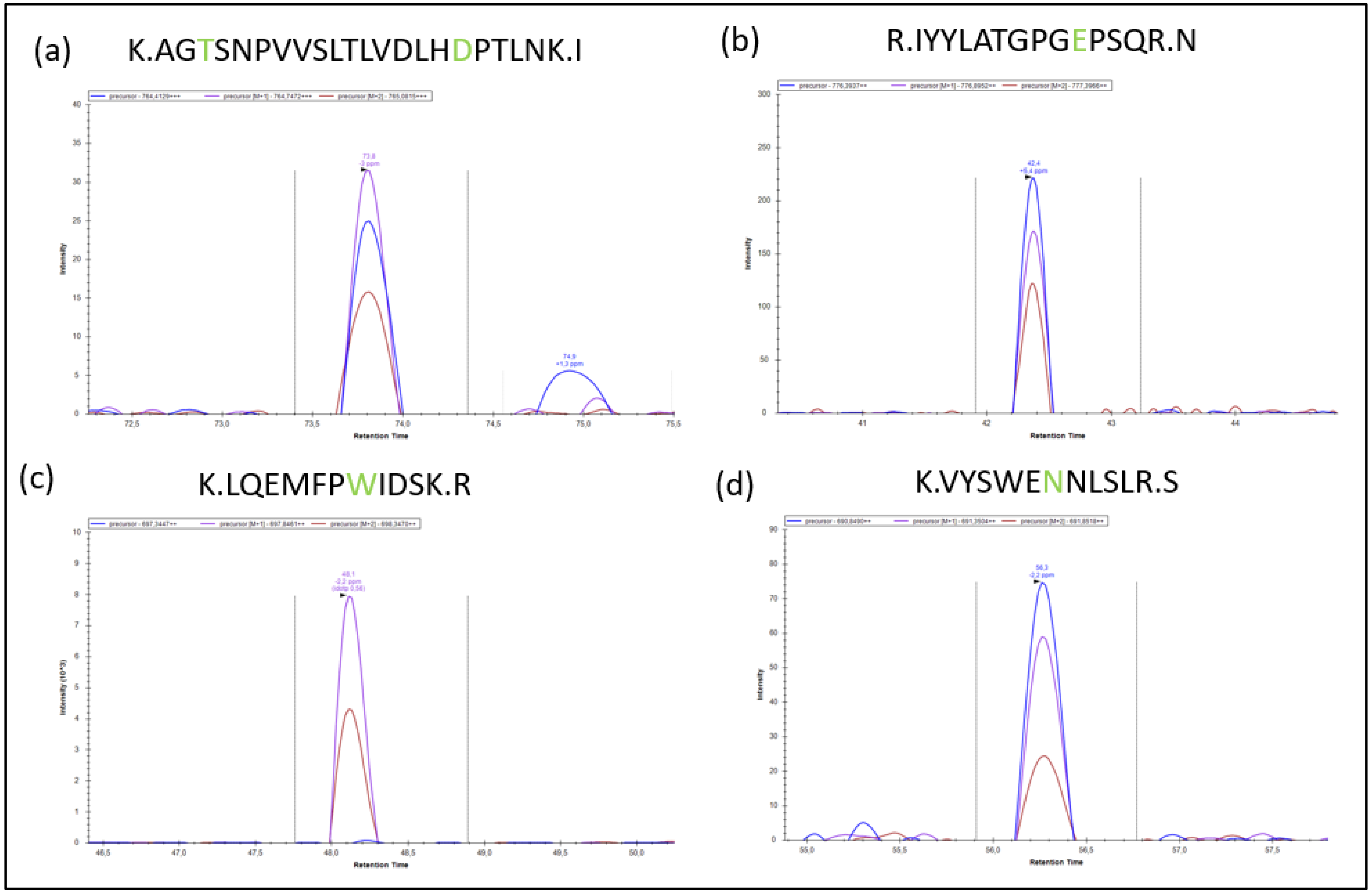

Figure S3 -- Skyline DIA data. A representative extracted precursor (top) and fragment ion (bottom) signal for the 4 peptides precursors. (a) K.AGTSNPVVSLTLVDLHDPTLNK.I, (b) R.IYYLATGPGEPSQR.N, (c) K.LQEMFPWIDSK.R, and (d) K.VYSWENNLSLR.S from DIA data acquired on VV samples digest using trypsin. The vertical lines on either side of the peak indicate the integration limits for the peak. The vertical blue line shows the retention time of the peptide identification included in the protein sequence. The mass measurement error and retention time of the most intense transition (fragment ion data) or isotopic peak (precursor data) for the peptide precursor are annotated above the chromatographic peak. Amino acids shown in green correspond to residues that differ in the newVespv3 sequence and the sequence from *V.basalis*. DIA, data-independent acquisition; VV, *Vespa velutina*.

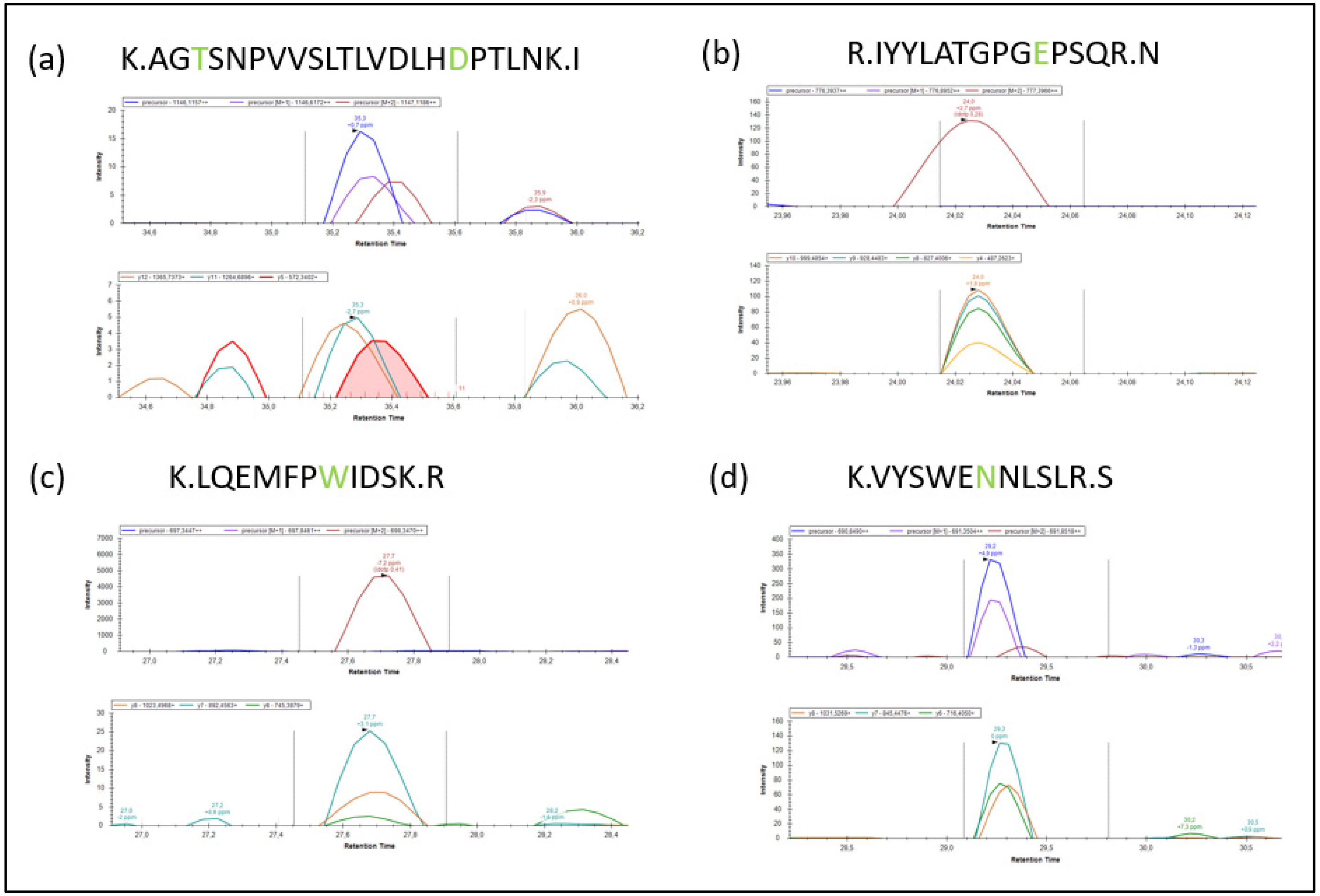

Supplement: Supplementary file 1 [file toxins-15-00656-s001.zip › toxins-2680620-supplementary.pdf]
